# Psychometric Analysis of Two Brief Versions of the CERQ in the Argentinean Population: CERQ-18 and CERQ-27

**DOI:** 10.11621/pir.2023.0102

**Published:** 2023-03-15

**Authors:** Sergio Dominguez-Lara, Luciana Moretti, Pablo Ezequiel Flores-Kanter, Roger Muñoz-Navarro, Leonardo A. Medrano

**Affiliations:** a ^Universidad^ de San Martín de Porres, Lima, Perú; b Pontificia Universidad Católica Madre y Maestra, República Dominicana; c Universidad Siglo 21, Argentina; d Consejo Nacional de Investigaciones Científicas y Técnicas (CONICET), Argentina; e Universitat de València, Spain

**Keywords:** Emotion, validity, reliability, cognitive regulation, short scale

## Abstract

**Background:**

Emotion Regulation (ER) involves any explicit or implicit process that may alter the emotion felt, its duration and expression, and is a transdiagnostic factor of vulnerability involved in the etiology and maintenance of different emotional disorders. The Cognitive Emotion Regulation Questionnaire (CERQ) assesses nine cognitive strategies involved in ER and is a valuable tool. Its popularity and wide use led to the development of two abbreviated versions: a version with 18 items (two items per factor) and a 27-item version (three items per factor).

**Objective:**

To analyze the psychometric properties of both versions in the Argentinean population.

**Design:**

The research design was instrumental. The factor structure of the CERQ-18 and CERQ-27 as well as the reliability of the scores and the construct of each dimension were evaluated. In addition, we gathered validity evidence for its relationship with other variables by associating the CERQ scores with Difficulties in Emotion Regulation Scale (DERS) scores.

**Results:**

The CERQ-18 presented more consistent evidence regarding its internal structure (adequate fit indices and factor loadings of moderate magnitude) and reliability. Given that the association of the two versions with the DERS is similar, we recommend that the 18-item version be used.

**Conclusion:**

The CERQ-18 has quite similar psychometric properties to the CERQ-27 in the general population of Argentina and the findings contribute to an understanding of its internal structure.

## Introduction

Emotions are adaptive responses that favor our survival; however, when their intensity, frequency or duration occurs inappropriately or excessively, they can affect our psychosocial functioning and harm our quality of life. Emotion Regulation (ER) has a transdiagnostic nature, since it appears to be associated with a wide variety of mental disorders (Aldao et al., 2010; Duarte et al., 2015; Potthoff et al. 2016; Sakakibara & Kitahara, 2016).

ER involves any explicit or implicit process that may alter the emotion felt, its duration and/or expression (Denny et al., 2009). Among the factors involved in ER, cognitive processes play a prominent role (Garnefski & Kraaij, 2007). Indeed, attention to circumstances and the cognitive interpretation of events will determine the type of emotion experienced and the modulation of the emotional response (Joormann et al., 2009; Muñoz-Navarro et al., 2021).

Within the strategies of cognitive regulation of emotion, Garnefski and Kraaij (2007) have distinguished nine: 1) *Self-Blame*: thoughts in which the person blames him/herself for what was experienced; 2) *Blaming Others*: thoughts of blaming environmental factors or other people for the unpleasant experience; 3) *Rumination:* reiterative thoughts about negative emotions and ideas associated with a stressful event; 4) *Catastrophizing:* thoughts that magnify the negative; 5) *Putting into Perspective:* thoughts that minimize the seriousness of a situation by emphasizing the relativity of what happened when compared to other events; 6) *Positive Refocusing*: thoughts about pleasant topics instead of focusing on the stressful event; 7) *Positive Reappraisal*: providing an alternative interpretation by seeking a positive connotation or meaning in terms of personal growth resulting from an event; 8) *Acceptance*: the cognitive process by which the individual stops trying to change a negative situation or the emotions that it caused and just experiences them; and finally, 9) *Refocus on Planning*: thinking about how to handle a problematic situation or what steps to take to resolve it. These nine strategies are the basis for the Cognitive Emotion Regulation Questionnaire (CERQ, Garnefski & Kraaij, 2007).

According to Medrano et al. (2016) the nine CERQ factors can be explained from the contributions of evolutionary psychology: that human beings evolved from the adoption of behaviors that favored their survival. Thus, the tendency to think excessively and repeatedly about negative events, or to pay greater attention to negative stimuli, favored the survival of our ancestors. These primitive and archaic cognitive patterns would be activated whenever a person detected a threat. These cognitive processes, being evolutionarily ancient, would involve subcortical brain structures, which would lead them to be automatic, simple, rapid, motivationally intense, and largely out of voluntary control. Such is the case for Rumination and Catastrophizing, for example.

On the other hand, during recent evolutionary history the human species developed more elaborate cognitive patterns. These new functions are based on cortical structures, which are more complex, rational, and motivationally more diffuse, which is why they favor a slower, analytical and controlled response by the organism, in addition to consuming high attentional resources. Developing an alternative interpretation to a negative event, accepting the situation, giving it a positive meaning, or planning concrete courses of action in the face of a negative event, requires more complex cognitive processes that demand conscious effort (Clore & Ortony, 2000; Dunbar & Shultz, 2007; Medrano et al., 2016), such as through Positive Reappraisal or Refocus on Planning. Based on this, we proposed to group cognitive strategies of emotion regulation into two broad categories: a) *automatic*, a primarily subcortical response (fast and difficult to regulate), and b) *elaborative*, a primarily cortical response (slow and analytical). Difficulties in emotion regulation are due to failures in the inhibition of automatic processes. For this reason, we hypothesized that automatic strategies (such as Catastrophizing or Rumination) are associated with greater difficulties in emotion regulation, while elaborative strategies (such as Positive Reappraisal or Refocus on Planning) present an inverse relationship with difficulties in emotion regulation.

The nine-factor model has been examined in several countries, such as Argentina, (Medrano et al., 2013), Iran (Abdi et al. 2012), Brazil (Schäfer et al. 2018), Portugal (Costa-Martins et al., 2016), France (d’Acremont & Van der Linden, 2007), Peru (Dominguez-Lara & Medrano, 2016), Spain (Domínguez-Sánchez et al., 2011), Turkey (Tuna & Bozo, 2012), the Netherlands (Garnefski et al., 2002), Arab countries (Megreya et al., 2016), Hungary (Miklósi et al., 2011), and China (Zhu et al., 2008). However, these studies focused on university samples, and studies with adults were less frequent (Ireland et al., 2017), senior adults (Carvajal et al., 2022) or clinical samples (McKinnon et al., 2020). The only studies with adult samples were found in the Netherlands (Garnefski & Kraaij, 2007), France (Jermann et al., 2006), and Romania (Perte & Miclea, 2011). In some studies, the oblique nine-factor structure achieved mainly poor statistical support with a confirmatory factor analysis (CFA), with poor factorial fit (Medrano et al., 2013; Tuna & Bozo, 2012; Zhu et al., 2008), and in some cases relatively low factor loadings were observed in some items (Dominguez-Lara & Medrano, 2016; Jermann et al., 2006; Medrano et al., 2013). This could be explained by the high interfactorial correlation between some factors (*e.g.*, Rumination and Catastrophizing), and in potential cross-loadings between items belonging to factors with moderate or high associations with each other, i.e., in possible misspecifications in the model (Dominguez-Lara & Merino-Soto, 2018b; Saris et al., 2009). On the other hand, in those studies with an exploratory approach, it was observed that two dimensions merged (Perte & Miclea, 2011), or that items loaded on other factors, but not in the original one (Adbi et al., 2012), which is feasible, considering that exploratory factor analysis does not restrict item grouping to a given factor; it is possible that this reorganization reflects the true structure of the construct in the countries where the CERQ was analyzed.

The CERQ has proven to be a valuable tool in diverse professional applications. Its use at the clinical (Huh et al., 2017), organizational (Castellano et al., 2019) and educational (Vinter et al., 2020) levels allows the assessment of risk and protective factors in the response to emotionally conflictive or stressful situations (Garnefski & Kraaij, 2007). It is also a widely used instrument to assess the effectiveness of psychological interventions (Bernard & Walburg, 2020; Hamid et al., 2018).

Its popularity and wide use led to the development of two abridged versions. First, Garnefski and Kraaij (2006) developed a short version of the CERQ with 18 items, so that each of the original nine scales is assessed by two items. Despite this reduction in the number of items, the questionnaire maintains the factor structure and adequate psychometric properties (alpha coefficients between .68 and .81). However, a descriptive approach (corrected item-test correlation) was used to select the items of this version; to consolidate the internal structure, the Principal Components Analysis (PCA) with varimax rotation was used, and although the factor loadings were high, they may be inflated because the PCA includes specificity within the factor loadings. It is worth mentioning that the Little Jiffy — a combination of the PCA, varimax rotation, and Kaiser’s rule — is disregarded (Lloret-Segura et al., 2014).

In that sense, a new brief version was developed with methods more consistent (Dominguez-Lara & Merino-Soto, 2015), and the items were selected considering empirical criteria (items with high factor loadings in preceding studies that had used a confirmatory approach) and theoretical criteria (items whose content was more compatible with the target strategy). The oblique nine-factor structure was supported by CFA, and later the findings were reaffirmed in a new study, where evidence was also obtained of its factorial equivalence with the 36-item version, as well as its association with depression and anxiety (Dominguez-Lara & Merino-Soto, 2018). The CERQ-18 performed well there, because it considers items with greater theoretical and empirical convergence with the construct, which is reflected in a more consistent version.

Considering that the psychometric literature discourages the measurement of latent variables with only two items, Holgado-Tello et al. (2018) developed an abbreviated version with 27 items, designed to assess the nine original dimensions of the model with three items per factor. The items were selected under an empirical criterion, by considering the items with the highest factor loadings after performing a CFA, although without specifying a minimum magnitude. From comparison of the two versions, Holgado-Tello et al. (2018) argue that the 27-item version is more appropriate for the specific assessment of the nine emotional regulation strategies and that the 18-item version is more appropriate in situations that require a global rating of the emotional regulation profile.

However, it is important to note that the results obtained suggest that both abbreviated versions have adequate psychometric properties and present similar test-criterion relationship evidence, so the superiority of one version over the other is debatable.

In light of the increasing use of brief instruments in the international literature, the present article aims to analyze the psychometric properties of the two brief versions of the CERQ in the Argentinean population, evaluating the factor structure of the CERQ-18 and CERQ-27 as well as the reliability of the scores and the construct of each dimension. In addition, we gathered validity evidence for its relationship with other variables by associating the CERQ scores with the Difficulties in Emotion Regulation Scale (DERS) scores, as was done in similar studies (Ireland et al., 2017). Direct and significant relationships were expected to be obtained between the DERS factors that assess difficulties in emotion regulation and the automatic strategies assessed by the CERQ (Catastrophizing, Rumination, Self-Blame, and Blaming Others). On the other hand, inverse and significant relationships were expected to be obtained between DERS scores and the CERQ-assessed elaborative strategies (Positive Reappraisal, Positive Refocusing, Refocus on Planning, Acceptance, and Putting into Perspective).

## Methods

### Design

The research design was instrumental (Ato et al., 2013), examining the psychometric properties of two brief versions of CERQ.

### Participants

The sample consisted of 800 Argentinean adults (60.1% women; 39.9% men; M_age_ = 30.10 years; SD_age_ = 12.99) selected from a non-probabilistic accidental sampling. Regarding the level of instruction: 0.4% had only completed primary school, 31.3% had incomplete secondary school, 14.4% had completed secondary school, 27.9% had incomplete tertiary or university studies, 21.0% had completed university studies, and 4.4% had postgraduate studies. Regarding participants’ occupations, 58.63% were employees, 31% students, 2.75% housekeepers, 2% unemployed, 0.5% retirees, and 5.13% did not answer. Regarding geographic distribution, most of the sample comes from Córdoba (54%) and Buenos Aires (36%). The remaining 10% is distributed in other regions of the country.

### Procedure

#### Questionnaire

The Cognitive Emotional Regulation Questionnaire (CERQ; Garnefski et al., 2002) consists of 36 items and has five polytomous response options ranging from almost never (1) to always (5). The items are grouped into nine strategies: *Rumination, Catastrophizing, Self-Blame, Blaming Others, Putting into Perspective, Acceptance, Positive Refocusing, Positive Reappraisal*, and *Refocus on Planning.* The Spanish version validated in Argentina (Medrano et al., 2013) was used. The 18-item version (Dominguez-Lara & Merino-Soto, 2018a) and the 27-item version (Holgado-Tello et al., 2018) were analyzed.

The Difficulties in Emotion Regulation Scale (DERS; Gratz & Roemer, 2004) is a 36-item self-report measure which has four dimensions: *Lack of Emotional Acceptance, Interference in Goal-Directed Behavior, Impulse Control Difficulties, Lack of Emotional Awareness, Lack of Emotional Clarity,* and *Limited Access to Emotion Regulation Strategies*. Participants are asked to indicate how often the items apply to themselves using a five-point Likert scale, with 1 = *almost never* (0–10%), 2 = *sometimes* (11%–35%), 3 = *about half the time* (36%–65%), 4 = *most of the time* (66%–90%), and 5 = *almost always* (91%–100%). Higher scores on each subscale indicate greater difficulties in emotion regulation. Preliminary evidence in Argentina (Medrano & Trógolo, 2014, 2016) suggests good psychometric properties of DERS, with adequate reliability for all subscales (alpha coefficients ranging from .70 to .87), except for limited access to the emotion regulation strategies subscale (Cronbach’s alpha = .54) and concurrent validity with personality measures.

All participants included in the study received an informed consent statement highlighting the voluntary nature of participation, and the questionnaires were completed anonymously. Of the total number of participants, 54.3% responded to the online instruments through the Google Form platform and 45.7% responded in person, on paper. No statistically significant differences were observed between the two samples in terms of CERQ scores (t_[764]_ = .76; *p* = .44).

#### Data Analysis

To evaluate the internal structure of the CERQ-18 and CERQ-27, a confirmatory factor analysis (CFA) was performed, specifying the oblique nine-factor model, which has been extensively examined in previous studies. For this purpose, the WLSMV extraction method was used, taking as a basis the matrix of inter-item polychoric correlations.

The validity evidence in relation to its internal structure was evaluated considering three perspectives. The first was based on the magnitude of the most frequent fit indices used in the literature such as the CFI (> .90; McDonald & Ho, 2002), the RMSEA (< .08; Browne & Cudeck, 1993), and the WRMR (< 1; DiStefano et al., 2018).

The second perspective was based on analysis of potential misspecification associated with cross-loadings (Saris et al., 2009) with a specialized module (Dominguez-Lara & Merino-Soto, 2018b).

The third perspective was based on the empirical differentiation of the dimensions. This is a key aspect in the construction of multidimensional instruments, since in addition to the conceptual differentiation between the factors, there must also be empirical differentiation, and although there may be elements in common between them, each must retain its individuality so that the findings can be interpreted in terms of the desired factor. One aspect that can give evidence of such differentiation is the comparison of the AVE (average variance extracted) of a factor, with the squared interfactor correlation (ϕ^2^; variance shared between factors), where the average variance extracted per factor (> .50; Fornell & Larcker, 1981) was expected. Mplus software version 7.0 (Muthén & Muthén, 1998–2015) was used.

To estimate construct reliability, the ω coefficient was used (> .70; Hunsley & Marsh, 2008). The reliability of the scores was estimated using the average inter-item correlation (*r_ii_*) since the dimensions have few items (2, 3, and 4 items), expecting magnitudes greater than .40 (Clark & Watson, 1995). Finally, a comparison was made between the *r_ii_* of each dimension among the three versions (CERQ, CERQ-27, and CERQ-18) with the *q* statistic expecting magnitudes smaller than .10 (Cohen, 1992) to conclude that the variation in reliability is not significant.

The equivalence between the brief versions, the CERQ-27 and CERQ-18, was analyzed separately. A procedure that corrects correlations between variables with items in common (Levy, 1967) was used, after which magnitudes above .70 are expected to conclude on the equivalence between versions (Putnam & Rothbart, 2006). Prior to this, skewness and kurtosis were assessed, expecting magnitudes between –2 and 2 (Gravetter & Wallnau, 2013).

Finally, the bivariate correlations between the CERQ factors of each brief dimension, CERQ-27 and CERQ-18, and the DERS factors were analyzed, considering magnitudes above .20 as significant (Ferguson, 2009). IBM SPSS 20 software was used to perform these analyses. In the same way, the correlations between the DERS dimensions with the CERQ-27 and the DERS with the CERQ-18 were compared using the *q* statistic. The absolute value of the average correlation between CERQ and DERS was considered for comparisons.

## Results

Regarding the analysis of validity evidence in relation to internal structure and reliability, all fit indices of the nine-factor model were adequate (CFI = .974; RMSE A = .046 [CI90% .037, .055]; WRMR = .768). The AVE was also higher than the shared variance between factors, except for the indicators associated with the relationship between Positive Reappraisal [E7] and Refocus on Planning [E8] (Table 1). However, 14 possible misspecifications associated with omitted cross-loadings were found. Regarding descriptive statistics, skewness and kurtosis reached adequate values.

**Table 1:** Parameters of the Oblique Nine-Factor Model — CERQ-18

	E1	E2	E3	E4	E5	E6	E7	E8	E9
Item 9^a^	.756								
Item 29	.769								
Item 17		.601							
Item 33		.874							
Item 2			.707						
Item 16			.850						
Item 15				.632					
Item 27				.800					
Item 10					.822				
Item 35					.836				
Item 11						.591			
Item 20						.883			
Item 23							.750		
Item 31							.915		
Item 13								.736	
Item 30								.661	
Item 14									.754
Item 24									.843
ω	.735	.713	.757	.681	.815	.714	.822	.657	.780
AVE	.581	.563	.611	.520	.687	.565	.700	.489	.640
E1	1	.001	.057	.051	.227	.031	.060	.016	.001
E2	.026	1	.024	.327	.192	.005	.010	.038	.001
E3	–.239	.154	1	.005	.058	.177	.213	.276	.039
E4	.225	.572	.068	1	.575	.000	.004	.067	.077
E5	.476	.438	–.240	.758	1	.016	.136	.009	.063
E6	–.177	.072	.421	–.016	–.127	1	.266	.158	.126
E7	–.245	.098	.462	–.066	–.369	.516	1	.567	.449
E8	–.127	.195	.525	.259	–.094	.397	.753	1	.311
E9	.032	–.030	.197	–.278	–.250	.355	.670	.558	1
M	4.139	6.054	7.834	6.365	4.980	7.282	7.451	8.311	6.385
SD	1.962	2.115	1.953	2.210	2.399	2.131	2.223	1.700	2.318
g_1_	0.684	–0.074	–0.824	–0.052	0.495	–0.598	–0.704	–1.050	–0.172
g_2_	–0.172	–0.581	0.276	–0.703	–0.725	–0.205	–0.242	0.911	–0.807

The analysis carried out with the CERQ-27 indicates that the fit indices of the nine-factor model had less favorable results than with the CERQ-18. Two indicators were not adequate (CFI = .893; WRMR = 1.394), while only the RMSE A was adequate (RMSE A = .068 [CI 90% .063, .073]). Similar to the CERQ-18, the AVE was greater than the shared variance between factors, except for the indicators associated with the relationship between Positive Reappraisal [E7] and Refocus on Planning [E8] (Table 2). However, 56 possible misspecification errors associated with omitted cross-loadings were found. Regarding descriptive statistics, skewness and kurtosis reached adequate values.

**Table 2:** Parameters of the Oblique Nine-Factor Model — CERQ-27

	E1	E2	E3	E4	E5	E6	E7	E8	E9
Item 9^a^	.707								
Item 29	.814								
Item 36	.678								
Item 1		.652							
Item 17		.694							
Item 33		.753							
Item 2			.687						
Item 16			.786						
Item 32			.603						
Item 3				.504					
Item 15				.656					
Item 27				.786					
Item 10					.811				
Item 22					.549				
Item 35					.853				
Item 11						.646			
Item 20						.707			
Item 34						.721			
Item 12							.685		
Item 23							.732		
Item 31							.850		
Item 13								.778	
Item 19								.637	
Item 30								.704	
Item 4									.780
Item 14									.771
Item 24									.796
ω	.778	.743	.736	.690	.789	.734	.802	.750	.826
AVE	.541	.491	.485	.434	.562	.479	.576	.502	.612
E1	1	.000	.045	.040	.299	.005	.030	.004	.002
E2	.009	1	.052	.371	.212	.018	.002	.031	.007
E3	–.213	.229	1	.046	.040	.250	.303	.308	.082
E4	.201	.609	.215	1	.476	.025	.002	.169	.057
E5	.547	.460	–.199	.690	1	.002	.099	.009	.084
E6	–.074	.133	.500	.158	–.042	1	.372	.228	.218
E7	–.174	.046	.550	.048	–.315	.610	1	.696	.480
E8	–.060	.175	.555	.411	–.094	.477	.834	1	.250
E9	.041	–.082	.287	–.238	–.289	.467	.693	.500	1
M	6.249	8.906	11.479	10.119	6.906	11.004	11.599	12.234	9.746
SD	2.715	2.858	2.661	2.874	3.152	2.954	2.885	2.482	3.283
g_1_	0.679	–0.009	–0.701	–0.266	0.622	–0.589	–0.729	–1.010	–0.217
g_2_	0.150	–0.380	0.171	–0.341	–0.385	–0.166	–0.062	0.961	–0.739

Regarding the construct reliability of the CERQ-18 and CERQ-27, most of the dimensions present acceptable magnitudes, although in relation to the reliability of the scores, the magnitudes are acceptable (*r_ii_* > .40; Table 3, Part 1). Regarding the comparison of the reliability of scores (*r_ii_*) between versions, the CERQ-27 presents more favorable indicators than the CERQ only in Acceptance, while the CERQ-18 surpasses the CERQ in Blaming Others, Self-Blame, Acceptance, Catastrophizing, Putting into Perspective, and Positive Reappraisal. Regarding the comparison between the brief versions, CERQ-18 outperforms CERQ-27 in Acceptance, Catastrophizing, and Positive Reappraisal (Table 3, Part 2). Regarding the equivalence between long and short versions, the corrected association between CERQ and its short versions is marginal, and the correlation is equivalent between CERQ and its short versions (*q* < .10; Table 3, Part 3).

**Table 3:** Three Versions of CERQ: Reliability and Equivalence between Versions

	Part 1						Part 2			Part 3				
	CERQ		CERQ-27		CERQ-18		Comparison of versions in terms *r_ii_*			CERQ/CERQ-27		CERQ/CERQ-18		*q*
	α	*r_ii_*	α	*r_ii_*	α	*r_ii_*	q36.27	q36.18	q27.18	*r*	*r_corrected_*	*r*	*r_corrected_*	
E1	.67	.34	.70	.44	.65	.48	.09	.12	.04	.92	.69	.86	.66	.02
E2	.69	.36	.68	.42	.63	.46	.05	.09	.04	.95	.69	.88	.66	.02
E3	.53	.22	.64	.38	.68	.52	.14	.26	.12	.91	.60	.81	.61	.00
E4	.68	.35	.64	.37	.62	.45	.02	.09	.07	.94	.66	.85	.63	.02
E5	.71	.38	.72	.46	.76	.62	.07	.19	.12	.95	.72	.87	.72	.00
E6	.66	.33	.66	.39	.61	.44	.06	.10	.04	.94	.66	.87	.63	.02
E7	.74	.42	.71	.45	.76	.61	.03	.15	.12	.97	.73	.90	.75	.01
E8	.66	.33	.67	.40	.55	.38	.07	.05	.01	.95	.67	.86	.61	.04
E9	.84	.57	.78	.54	.72	.56	.03	.01	.02	.98	.81	.94	.79	.01

*Note: E1 = Blaming Others, E2 = Self-Blame; E3 = Acceptance; E4 = Rumination; E5 = Catastrophizing; E6 = Putting into Perspective; E7 = Positive Reappraisal; E8 = Refocus on Planning; E9 = Positive Refocusing; r_ii_: average inter-item correlation; q_n.m_: comparison of r_ii_ between n-items version and m-items version.*

In relation to their association with other variables, the relationships among the nine CERQ factors, both the CERQ-27 and CERQ-18, and the DERS dimensions were analyzed, and verified with the CERQ factors called automatic and elaborative. The results show significant correlations in most cases (> .20; Table 5). Specifically, the strategies Self-Blame, Blaming Others, Rumination, and Catastrophizing show the strongest and most positive relationships with the different difficulties in emotion regulation. In general, an association between difficulties in emotion regulation and automatic cognitive strategies is corroborated.

On the other hand, a strong relationship between elaborative strategies and difficulties in emotion regulation is not corroborated. The strategies of Acceptance, Putting into Perspective, Positive Reappraisal, Refocus on Planning, and Positive Refocusing are low (*r* < .20), and most of them do not reach significance. A significant relationship was only observed between the elaborative strategies with the lack of emotional awareness and lack of emotional clarity scales. Another aspect to highlight is that the relationship between automatic and elaborative strategies is weak. These results therefore suggest a certain independence; thus, the predominance of automatic strategies of emotion regulation may or may not coexist with elaborative strategies.

On the other hand, the intensity of the correlation between cognitive strategies and emotion regulation difficulties is similar between both versions (q < .10; Table 4), but of weak magnitude.

**Table 4:** Correlation between DERS and the Short Versions of CERQ

	D1	D2	D3	D4	D5	D6	*|r_average_* **|**
E1_CERQ_	.326	.317	.336	.008	.208	.249	.241
E1_CERQ18_	.313	.300	.349	–.001	.146	.229	.223
E1_CERQ27_	.303	.290	.348	–.022	.164	.237	.220
E2_CERQ_	.390	.543	.295	.074	.109	.390	.300
E2_CERQ18_	.281	.223	.082	.077	.067	.223	.159
E2_CERQ27_	.339	.275	.124	.045	.063	.271	.186
E3_CERQ_	.159	.048	.031	.108	.129	.087	.094
E3_CERQ18_	.014	.001	–.073	.231	.150	–.036	.048
E3_CERQ27_	.054	.021	–.023	.219	.144	.018	.072
E4_CERQ_	.407	.274	.233	.137	.105	.317	.246
E4_CERQ18_	.269	.242	.200	.122	.091	.254	.196
E4_CERQ27_	.284	.230	.160	.241	.089	.248	.209
E5_CERQ_	.286	.239	.319	–.117	.050	.258	.173
E5_CERQ18_	.310	.338	.407	–.120	.067	.328	.222
E5_CERQ27_	.319	.284	.371	–.113	.040	.300	.200
E6_CERQ_	.222	.119	.097	.172	.137	.117	.144
E6_CERQ18_	.176	.121	.069	.157	.177	.124	.137
E6_CERQ27_	.202	.128	.063	.155	.157	.115	.137
E7_CERQ_	–.006	–.047	–.038	.294	.161	–.076	.048
E7_CERQ18_	–.032	–.050	–.040	.243	.107	–.082	.024
E7_CERQ27_	.009	–.028	–.019	.273	.151	–.057	.055
E8_CERQ_	.123	.099	–.040	.248	.176	.046	.109
E8_CERQ18_	.100	.000	–.064	.229	.184	–.004	.074
E8_CERQ27_	.109	.057	–.032	.263	.180	.017	.099
E9_CERQ_	.037	–.155	–.082	.027	.052	–.075	–.033
E9_CERQ18_	.072	–.119	–.029	–.007	.051	–.005	–.006
E9_CERQ27_	.057	–.135	–.055	.031	.054	–.063	–.019

*Note: E1 = Blaming Others, E2 = Self-Blame; E3 = Acceptance; E4 = Rumination; E5 = Catastrophizing; E6 = Putting into Perspective; E7 = Positive Reappraisal; E8 = Refocus on Planning; E9 = Positive Refocusing; D1 = Lack of Emotional Acceptance; D2 = Interference in Goal-Directed Behavior; D3 = Impulse Control Difficulties; D4 = Lack of Emotional Awareness; D5 = Lack of Emotional Clarity; D6 = Limited Access to Emotion Regulation Strategies, ^a^: in all cases, q coefficient compares CERQ-18 and CERQ-27.*

## Discussion

Understanding the ability to functionally regulate emotions is a key factor in understanding the psychological processes of health and disease (Cano-Vindel et al., 2016). Different psychological disorders, such as anxiety disorders and depression, are significantly related to emotion regulation styles. The CERQ is one of the most widely used instruments to assess cognitive factors involved in emotion regulation (Medrano et al., 2016); however, the length of the instrument may limit its use in professional practice. Due to the advantages of short questionnaires, two short versions of the CERQ have recently been developed with 18 and 27 items, with respectively two and three items per strategy. As Santisteban-Requena (2009) points out, the validity and reliability of a test may be affected as the length of the instrument is altered, so it would be necessary to evaluate empirically whether the short versions still adequately measure the construct.

The results obtained in the present study corroborate the adequate psychometric properties of the CERQ-18 in the Argentinean population, whereas in relation to the CERQ-27 the fit indices were not favorable and a high number of misspecifications were found that threaten the validity evidence. Indeed, the psychometric properties observed are acceptable and similar to those reported for the CERQ, but in some cases the reliability indicators evaluated with the average inter-item correlation was higher in the CERQ-18 with respect to the CERQ-27 and CERQ. In comparison to the 36-item version, in the case of the CERQ-18 the evidence of validity and reliability has been investigated by few studies. Consistent with this background (Dominguez-Lara & Merino-Soto, 2018a; Ireland, et al. 2017; Lee et al. 2020), evidence in favor of a correlated nine-factor structure is found in the present work.

Regarding the observed association between dimensions, the automatic factors of Catastrophizing, Rumination, and Self-Blame overlap considerably in both versions, as well as within the elaborative processes, the factors of Positive Reappraisal, Refocus on Planning, and Positive Refocusing. These results are consistent with some of the reported antecedent studies (*e.g.*, Ireland et al., 2017), which go so far as to posit the existence of two underlying factors (Domínguez-Sanchez et al., 2013; Perte & Miclea, 2011). As suggested by Thompson (1997), such a conceptual and empirical approach may lead one to think about the existence of higher-order factors, suggesting the possibility of an alternative model that contemplates the presence of second-order factors. However, it was not possible to evaluate the fit of an alternative hierarchical model of two higher order factors, because they were under-identified. That is, the number of indicators with the 18-item version is not sufficient to examine the fit of such a model.

Regarding the evidence of validity with external sources, the relationships between the nine CERQ factors of the 18-item and 27-item versions and the DERS dimensions were analyzed. In the first instance, the hypothesized relationships between the automatic factors of the CERQ and the difficulties in emotion regulation were verified. Specifically, it was observed that high scores on the factors of Self-Blame, Blaming Others, Rumination, and Catastrophizing were associated with impulse control difficulties, limited access to emotion regulation strategies, and interference in goal-directed behaviors. These results are consistent with those reported in previous research (Medrano et al., 2016; Muñoz-Navarro et al., 2021). The magnitude of the correlations is similar between the dimensions of the DERS and the two versions of the CERQ-18.

The relationship between elaborative cognitive strategies and difficulties in emotion regulation turned out to be more complex. The relationships were lower than expected (*r* < .20 and mostly non-significant). These findings could be attributed to either a) the short version of the CERQ does not adequately measure the elaborative factors, or b) the elaborative factors are not significantly associated with difficulties in emotion regulation. Considering previous research, the second option is more plausible. In fact, there is currently a debate about the role of elaborative processes and their role in the development of emotional disorders. Evidence suggests that it is automatic processes that are involved in the etiology and maintenance of emotional disorders, while elaborative processes would only play a role in modulating the automatic processes (Medrano et al., 2016). Thus, elaborative processes would not be directly related to difficulties in emotion regulation, but would mediate the impact of automatic processes.

It should also be noted that the relationship observed between automatic and elaborative strategies was weak. These results are consistent with previous research (Castellano et al., 2019; Dominguez-Lara & Medrano, 2016) and allow us to rule out the existence of an inverse relationship between the two types of processing. The use of elaborative strategies would not generate a decrease in automatic strategies, although it may possibly moderate their impact. It may happen that two people experience an automatic process (*e.g.*, Catastrophizing or Rumination), but in one case this process is modulated by an elaborative strategy (*e.g.*, reinterpretation) and in another case it is not. It would therefore be more useful to analyze profiles of cognitive regulation of emotions, rather than analyzing strategies independently and in isolation from each other. In fact, in a study by Trógolo and Medrano (2012), it was observed that when considering emotion regulation profiles with the DERS, greater predictive power was achieved than when considering each strategy in isolation.

A final aspect to note is the association between the elaborative strategies with the factors of lack of emotional awareness, and lack of emotional clarity. These results are consistent with previous research highlighting the role of emotional awareness as a preliminary step for the use of elaborative strategies (Price & Hooven, 2018). Thus, the lack of emotional clarity and awareness could be interpreted as a factor that hinders the use of elaborative strategies of emotional regulation.

Another important finding is that the shorter version, the CERQ-18, shows more consistent evidence regarding its internal structure (adequate fit indices and factor loadings of moderate magnitude) and reliability, in contrast to the CERQ-27, which presents weaker indicators. For that reason, and because the association of the two versions with the DERS is similar, it is advisable to use the 18-item version.

## Conclusion

The CERQ-18 has psychometric properties quite similar to those of the CERQ-27 in the general population of Argentina and the findings contribute to understanding its internal structure. However, regarding its association with other variables, a scale that evaluates dysfunctional aspects (dysregulation) was considered as an external criterion of validity, so it would be convenient to also use external criteria focused on positive variables (*e.g.*, well-being) in order to have more information to decide on one version or another.

Having a brief instrument properly adapted to the Argentinean adult population makes it easier to develop studies aimed at evaluating the role of cognitive regulation of emotions in different contexts (educational, clinical, and organizational). In addition to its use in research, the present investigation provides a useful input for the identification of people with difficulties in emotion regulation and for the evaluation of interventions aimed at promoting more adequate styles of emotion regulation. The use of short versions is recommended in situations where, for reasons of time or sample disposition, the administration of the longer version is not possible.

Finally, it would be advisable to explore the patterns of association of the items with the factors to which they do not theoretically belong (cross-loadings) by means of exploratory structural equation modeling (ESEM ; Asparouhov & Muthen, 2009). A hierarchical model could be analyzed under ESEM to provide further empirical support for the presence of second-order factors called automatic strategies and elaborative strategies.

## Limitations

The sample is adequate, although it was not strictly representative of the general population of Argentina. It would therefore be useful to expand the sample with participants from other regions of the country.
